# Depth-Sensitive Optical Sensing for Non-Invasive Measurement of Human Muscle Activity

**DOI:** 10.3390/s26134172

**Published:** 2026-07-02

**Authors:** Kazunari Matsuo, D. S. V. Bandara, Hirofumi Nogami, Jumpei Arata

**Affiliations:** 1 Faculty of Engineering, Kyushu University, 744 Moto-o ka, Nishi-ku, Fukuoka 819-0395, Japan; kasado0253@gmail.com (K.M.); jumpei@mech.kyushu-u.ac.jp (J.A.); 2 Faculty of Engineering, Sojo University, 4-22-1, Ikeda, Nishi-ku, Kumamoto 860-0082, Japan; nogami@mec.sojo-u.ac.jp

**Keywords:** optical sensing, human–machine interface, depth-sensitive muscle activity, near-infrared, wearable sensors

## Abstract

Human muscle anatomy consists of multiple layers, each contributing to movement through complex patterns of activation. Conventional non-invasive sensing techniques, such as surface electromyography (sEMG) and mechanomyography (MMG), primarily capture aggregate muscle activity and provide limited depth-dependent information. As different movements may involve distinct combinations of superficial and deeper muscles, access to depth-dependent information could improve the discrimination of motion patterns that are difficult to distinguish using surface measurements alone. To address this limitation, we developed an optical sensor capable of depth-sensitive measurement using near-infrared light. The sensor comprises a light source and an array of photodetectors arranged at six source–detector distances (SDDs) ranging from 12 to 48 mm within a compact wearable module. Two experiments were conducted to evaluate the sensor. First, depth sensitivity was investigated using Monte Carlo simulations and phantom experiments, demonstrating distinct sensitivity profiles for different SDDs and providing preliminary evidence of depth-dependent sensing. Second, the sensor was attached to the forearm to measure signals during nine hand and wrist movements. Machine learning models were evaluated for motion classification, with Linear Discriminant Analysis (LDA) achieving the highest performance. Using all six SDD channels, an average classification accuracy of 87.5% was achieved across 10 subjects. An ablation study evaluating all 63 possible channel combinations further showed that classification performance improved systematically with the inclusion of multiple SDD channels, indicating that measurements obtained at different sensing depths provide complementary information for motion discrimination. These results demonstrate the feasibility of multi-SDD optical sensing for capturing depth-dependent physiological information and highlight its potential as a compact, non-invasive sensing approach for wearable human–machine interface applications.

## 1. Introduction

Wearable human–machine interfaces (HMIs) enable seamless interaction between humans and advanced technologies, including prosthetic hands, rehabilitation systems, exoskeletons, and virtual reality environments [1,2]. To achieve intuitive control, many HMIs rely on biosignals that reflect user intent. Common non-invasive sensing modalities include surface electromyography (sEMG), mechanomyography (MMG), forcemyography (FMG), and electroencephalography (EEG) [3,4,5,6,7]. While invasive approaches such as targeted muscle reinnervation (TMR) and intramuscular EMG provide improved selectivity of individual muscle activity, their clinical complexity limits their applicability in wearable systems [8,9,10]. Consequently, there remains a need for non-invasive sensing approaches capable of providing more discriminative information about underlying muscle activity.

Among non-invasive methods, sEMG is widely used for motion recognition in rehabilitation robotics and prosthetic control [11,12]. However, it suffers from limited spatial selectivity due to signal crosstalk, where electrical activity from multiple muscles overlaps [13,14]. This limitation is particularly significant in anatomically complex regions such as the forearm, where superficial and deep muscles may contribute simultaneously to multiple movements. As a result, the inability to distinguish depth-dependent muscle activity can lead to ambiguity in motion interpretation, reducing the accuracy and reliability of human–machine interface systems.

Alternative non-invasive techniques, including MMG and FMG, measure muscle activity through mechanical vibrations and volumetric changes, respectively [5,6]. While these methods offer alternative sensing modalities, they are also influenced by signals from multiple muscle layers, limiting their ability to selectively capture depth-dependent information. More advanced methods, such as high-density EMG and multi-electrode modeling, improve motion estimation by increasing spatial resolution and incorporating anatomical constraints [15,16,17]. However, these approaches often require complex hardware configurations and increased computational cost, limiting their practicality for wearable applications.

Optical sensing techniques have recently emerged as a promising non-invasive alternative. Methods such as near-infrared spectroscopy (NIRS) and pulse oximetry leverage the high biological transparency of near-infrared light to probe subsurface tissue [18,19,20,21]. Unlike sEMG, which measures electrical signals that reach the skin surface, optical sensing emits light into the tissue and analyzes how it is scattered and absorbed by underlying structures, allowing sensitivity to different depths. Furthermore, the source–detector distance (SDD) is known to influence the depth sensitivity of diffuse optical measurements, with larger SDDs generally increasing sensitivity to deeper tissue regions [22,23].

Recent studies have explored optical approaches for muscle activity measurement. Shahmohammadi et al. [24] proposed a lightmyography-based system that detects tissue deformation through optical variations, while Xie et al. [25] developed a multi-channel NIRS system to monitor muscle activity via oxygen saturation. Although these studies demonstrate the feasibility of optical sensing for muscle activity monitoring, they do not explicitly exploit measurements acquired at multiple sensing depths and often require multiple sensing locations or more complex sensor configurations to capture information from different muscle groups.

Table 1 summarizes representative muscle sensing approaches. While existing methods provide effective motion recognition, they generally lack the ability to explicitly capture depth-dependent muscle activity within a compact wearable sensing module. This limitation motivates the development of the proposed multi-SDD optical sensing approach.

Thus, there remains a need for a compact, non-invasive sensing approach that can provide depth-dependent information at the hardware level while maintaining practical system complexity. To address this limitation, this study proposes a novel optical sensing approach that integrates multiple SDDs within a single sensor module. By leveraging the depth-dependent sensitivity of different SDDs, the proposed design enables simultaneous acquisition of signals influenced by different tissue depths without requiring multiple sensor units. This configuration introduces a simple yet effective means of incorporating depth-sensitive information into wearable systems, enabling improved discrimination of motions involving layered muscle structures.

To investigate the feasibility of this approach, the proposed sensor was evaluated through Monte Carlo simulations, phantom experiments, and forearm motion-classification experiments. The sensor design and experimental methodology are described in the following sections.

## 2. Materials and Methods

This section outlines the methodology of the proposed system, including the sensing principle, sensor design, and evaluation methods.

### 2.1. Measuring Principle

**Figure 1:**
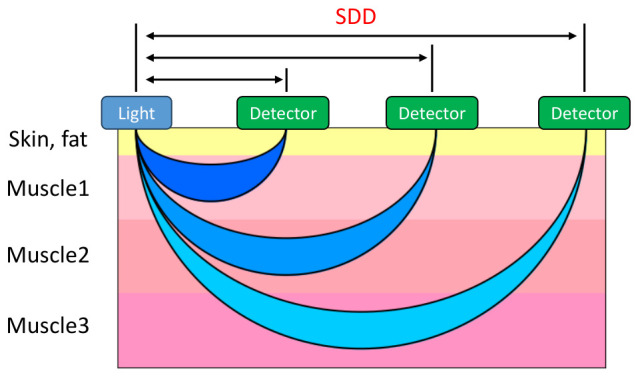
Concept of the optical sensor.

The measurement principle of the proposed optical sensor is illustrated in Figure 1. Near-infrared light is emitted into the body from a light source placed on the skin. As the light propagates through biological tissue, it is scattered and absorbed before being detected by photo-detectors positioned on the skin surface. The sensitivity of each source–detector pair follows a characteristic banana-shaped distribution theoretically, where the measurement depth increases with the SDD [21].

Variations in the detected light signal are influenced by muscle activity through several physiological and mechanical factors:

- Muscle displacement: Muscle contraction causes deformation and positional changes in the tissue, altering the optical path and affecting the detected light intensity [24].
- Tissue density changes: Structural changes in muscle fibers during contraction modify the optical properties of the tissue, influencing light scattering and reflection [26].
- Blood flow and oxygenation: Muscle activation leads to changes in local blood volume and oxygenation, which affect the absorption characteristics of near-infrared light [25].

In this study, the measured signal reflects the combined influence of these factors, and the proposed approach uses these variations to capture muscle activity with sensitivity controlled by the SDD.

### 2.2. Sensor Design

The sensor consists of a single light source and multiple photo-detectors positioned at different SDDs. Each source–detector pair exhibits a distinct sensitivity profile, with larger SDDs generally increasing sensitivity to deeper tissue regions. It is commonly accepted that the maximum penetration depth is approximately half the SDD [21]. Based on this principle, multiple SDDs were incorporated to obtain signals influenced by tissue activity at different depths.

**Figure 2:**
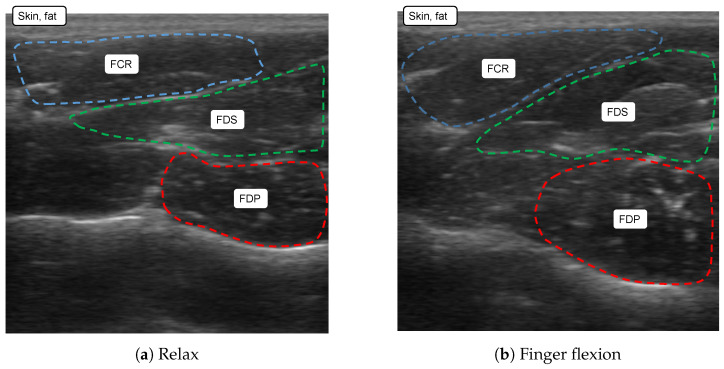
Forearm cross-section with ultrasound.

To determine appropriate SDD values, the musculoskeletal structure of the forearm was examined using a commercial ultrasound diagnostic device (EagleView, Guangzhou Sonostar Technologies Co., Ltd., Guangzhou, China). The forearm was selected due to its layered muscle structure. As shown in Figure 2, the flexor carpi radialis (FCR) is located in the superficial layer, the flexor digitorum superficialis (FDS) lies beneath it, and the flexor digitorum profundus (FDP) is located in the deepest layer. Measurements of muscle depth obtained from three subjects are summarized in Table 2. Based on these measurements, SDD values were selected such that their sensitivity profiles span these anatomical layers.

As illustrated in Figure 1, light reaching deeper regions also traverses superficial layers, meaning that signals measured at larger SDDs include contributions from multiple depths. Additionally, skin displacement due to muscle contraction may influence the detected signal. To mitigate these effects, signals from pairs of photo-detectors with similar SDD ranges are used for comparative analysis, with the aim of emphasizing depth-dependent differences in the measured signals.

Based on these considerations, the SDDs were selected as 12 mm, 16 mm, 28 mm, 32 mm, 44 mm, and 48 mm to provide sensitivity across superficial, intermediate, and deeper muscle regions.

### 2.3. Depth Simulation

Monte Carlo simulations (MCSs) were conducted to estimate the depth sensitivity associated with each SDD. The simulation framework is based on the Monte Carlo model of light propagation in tissue introduced by Wang et al. [27]. A simplified implementation was adopted to evaluate depth-dependent behavior, focusing on absorption and scattering effects. The simulated tissue consisted of three layers representing skin, subcutaneous adipose tissue, and muscle. The skin thickness was set to 1.4 mm and the adipose tissue thickness to 3.6 mm, while the muscle layer was modeled as a semi-infinite medium beneath 5.0 mm. These values were selected based on anatomical measurements obtained from ultrasound observations of the forearm. The anisotropy factor and refractive index were set to 0.90 and 1.33, respectively. Each detector was modeled as a square collection area of 3 mm × 3 mm positioned at the corresponding SDD.

Photons were emitted normal to the skin surface and propagated through the tissue, undergoing probabilistic absorption and scattering events. Each photon was initialized with a weight of 1, which decreased according to the absorption coefficient ($\mu_{a}$) and scattering coefficient ($\mu_{s}$) of the tissue layers (skin, subcutaneous fat, and muscle), based on reported optical properties [28]. The absorption coefficients used for skin, adipose tissue, and muscle were 0.33 cm^−1^, 1.07 cm^−1^, and 0.30 cm^−1^, respectively, while the corresponding scattering coefficients were 15.7 cm^−1^, 10.0 cm^−1^, and 6.67 cm^−1^. A total of $1\times 10^{9}$ photons were simulated and tracked until absorption or escape from the tissue model.

(1) $$w^{\prime }=w\cdot \left(1-\frac{\mu_{a}}{\mu_{a}+\mu_{s}}\right)$$

where *w* and $w^{\prime }$ represent the photon weight before and after absorption, respectively. Scattering events altered photon trajectories, and photons exiting the tissue within the detector region were recorded. The detector positions were defined according to the six SDD configurations (12 mm, 16 mm, 28 mm, 32 mm, 44 mm, and 48 mm) used in the fabricated sensor.

**Figure 3:**
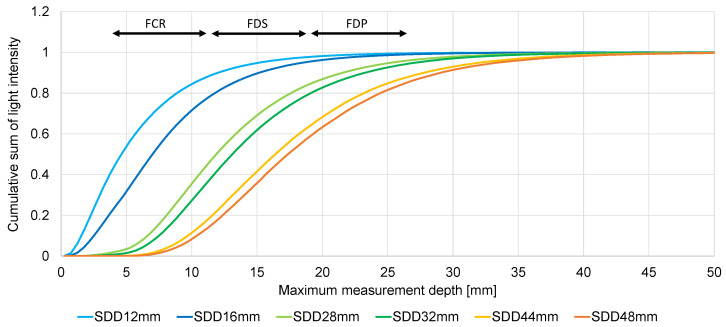
Simulation Results.

The depth sensitivity for each SDD was estimated by analyzing the distribution of photon penetration depths and the cumulative detected intensity. The results are shown in Figure 3, where the horizontal axis represents penetration depth and the vertical axis indicates cumulative light intensity.

For shorter SDDs (12 mm and 16 mm), the majority of detected photons originated from shallow regions, primarily within approximately 10 mm. For intermediate SDDs (28 mm and 32 mm), the sensitivity extended to deeper regions, with significant contributions observed in the range of approximately 5 mm to 20 mm. For longer SDDs (44 mm and 48 mm), the sensitivity further increased, with contributions extending to depths of approximately 10 mm to 30 mm.

These results indicate that increasing the SDD shifts the sensitivity of the measurement toward deeper tissue regions. Although the measurements are not confined to a single depth and include contributions from multiple layers, the variation in depth sensitivity across SDDs suggests that the detected signals contain depth-dependent information that can be exploited for distinguishing muscle activity in layered structure. It should be noted that the simulation was intended to provide a qualitative assessment of depth sensitivity associated with different SDDs rather than an anatomically complete model of forearm tissue. Therefore, the results should be interpreted as estimates of relative depth sensitivity rather than precise predictions of layer-specific signal contributions.

### 2.4. Sensor Fabrication

**Figure 4:**
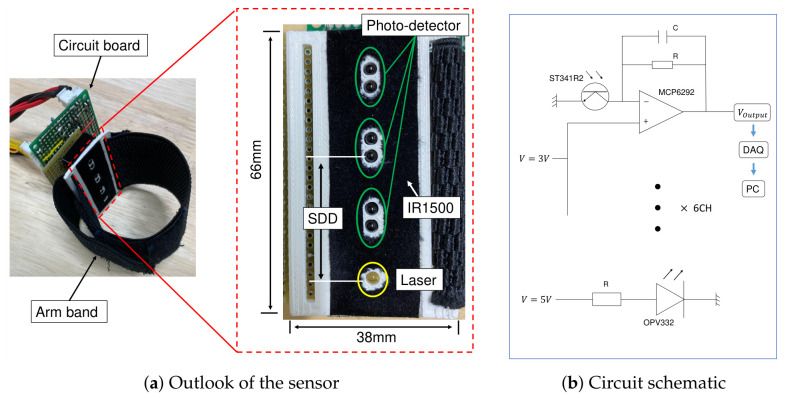
Sensor for measurement of deep layer muscle activity.

The developed sensor integrates a near-infrared light source with multiple photo-detectors arranged at different SDDs within a compact wearable configuration, as shown in Figure 4a. A near-infrared laser (OPV332, Optek Technology Inc., Carrollton, TX, USA) is used as the light source, while phototransistors (ST341R2, KODENSHI Corp., Kyoto, Japan) serve as light receivers. The light source is powered by a stabilized 5 V supply, operating at approximately 2.1 mW, and each phototransistor is driven by a 2 V supply. It operates at 850 nm in the near-infrared (NIR) region, where biological tissues exhibit high optical transparency, enabling deeper light penetration. The laser operated at approximately 2.1 mW, below the output limit of a Class 3R laser device. During operation, the light source remained in direct contact with the skin, minimizing the possibility of direct ocular exposure. These safety considerations were reviewed and approved as part of the institutional ethics review process, and no adverse effects or discomfort were reported by any participant during the experiments.

To reduce the influence of ambient light and surface reflections, the sensor surface is covered with a near-infrared absorbing cloth (IR1500, Koyo Orient Japan Co., Ltd., Saitama, Japan). Variations in detected light intensity are converted into electrical signals using a current-to-voltage conversion circuit based on an operational amplifier (MCP6292, Microchip Technology Inc., Chandler, AZ, USA), powered by a 6 V supply.

A low-pass filter with a cutoff frequency of 30 Hz was incorporated into the analog circuit to suppress high-frequency noise prior to digitization. This cutoff frequency was selected based on the expected low-frequency characteristics of optical signals associated with muscle activity, which are dominated by slow physiological processes such as tissue deformation and hemodynamic changes. Due to increased optical attenuation at longer SDDs, the detected signal amplitude decreases with distance. To compensate for this effect, the gain of each phototransistor channel is adjusted according to its SDD, enabling consistent acquisition of signals across different depths.

The analog voltage signals are digitized using a DAQ USB-5210 (National Instruments Corp., Austin, TX, USA) and recorded on a PC via LabVIEW software 19.0 for further processing.

### 2.5. Evaluation of the Measuring Depth

To evaluate the depth sensitivity of the developed sensor, an experiment was conducted using a layered ham phantom. Ham was selected as a biological tissue surrogate because it contains both muscle and adipose components and allows straightforward construction of layered structures with controllable thicknesses. Porcine tissues are widely used as biological phantoms in biomedical optics because they exhibit anatomical and physiological characteristics similar to those of human tissues while preserving realistic tissue heterogeneity and morphology [29]. This experiment provides a simplified and controlled assessment of depth-dependent optical behavior, rather than a full replication of in vivo physiological conditions.

**Figure 5:**
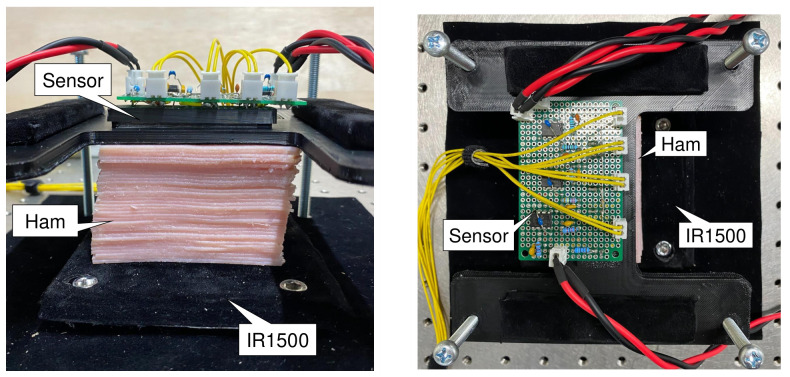
Experimental setup for evaluation of the measurement depth.

The experimental setup is shown in Figure 5. The sensor was fixed at the bottom, the layered phantom was placed above it, and a near-infrared absorbing cloth (IR1500) was positioned on top. Near-infrared light was emitted into the phantom, and the returned light intensity was measured by photo-detectors with different SDDs.

When the thickness of the phantom was smaller than the effective penetration depth associated with a given SDD, a portion of the light reached the absorbing cloth and was attenuated, resulting in a decrease in detected intensity. Conversely, when the phantom thickness exceeded the effective penetration depth, most photons were scattered within the medium and returned to the detectors, leading to relatively stable signal levels. Therefore, the range over which the detected light intensity varies with thickness provides an estimate of the depth sensitivity corresponding to each SDD.

The phantom consisted of up to 36 layers, corresponding to a maximum thickness of approximately 45 mm. The number of layers was decreased incrementally to zero while recording the signal from all six SDD channels, and then increased again to the maximum thickness. This process was repeated three times, and the averaged results were used for analysis.

### 2.6. Forearm Motion Classification

The performance of the developed sensor was evaluated for classifying multiple forearm motions, providing a practical assessment of its ability to capture depth-dependent muscle activity patterns for human–machine interface applications. Nine healthy male and one healthy female subjects participated in this experiment.

The sensor was attached to the flexor side of each subject’s forearm using an adjustable armband. To ensure consistent placement across subjects, the mounting position was determined relative to the forearm length, positioning the sensor over the central region of the flexor muscle group. This approach aimed to align the sensor with comparable anatomical locations across participants.

The target muscles included the FCR, FDS, FDP, and pronator teres (PT), as shown in Figure 2. These muscles span superficial to deep layers within the forearm. Nine common hand movements were performed: Relax, Finger flexion (FF), Finger extension (FE), Wrist flexion (WF), Wrist extension (WE), Radial deviation (RD), Ulnar deviation (UD), Pronation (PRO), and Supination (SUP). Table 3 summarizes the primary muscles involved in each movement. Agonist muscles are denoted by ∘, indicating muscles expected to contribute directly to the intended movement, whereas antagonist muscles are denoted by •, indicating muscles that oppose the movement but may still exhibit activation for joint stabilization and coordination.

Each movement was performed for 10 s per trial, followed by 10 s of rest. A full sequence of all movements constituted one set, and each set was repeated 15 times per subject, with a one-minute rest interval between sets. This protocol was designed to ensure sufficient data collection while reducing fatigue effects and maintaining consistency across trials.

All experimental procedures adhered to the ethical principles outlined in the Helsinki Declaration of 1975, as revised in 2000. Prior to participation, informed written consent was obtained from all subjects after a detailed explanation of the experimental protocol. The study was approved by the Institutional Ethical Review Committee (Approval No. 2024-5).

#### 2.6.1. Preprocessing

The light intensity signals were sampled at 1000 Hz from six SDD channels. To focus on the steady-state phase of each motion, the central 7 s of data were extracted for analysis, excluding the first and last 1.5 s to avoid transient effects during motion initiation and termination. This resulted in approximately 7000 samples per channel for each motion segment. Each time sample consisted of six simultaneously acquired SDD measurements and was treated as an independent input vector for classification.

Prior to each measurement session, a calibration recording was acquired and used to compensate for channel-dependent baseline offsets in the detected optical signals. The recorded signals were further processed using a digital low-pass filter with a cutoff frequency of 30 Hz to reduce residual noise and ensure consistent signal quality across channels. This cutoff was selected based on the low-frequency characteristics of optical signals associated with muscle activity, which are primarily influenced by slow physiological processes such as tissue deformation and hemodynamic changes. The use of both analog and digital filtering ensures suppression of high-frequency noise at the acquisition stage and refinement of signal quality during post-processing. A moving average filter with a window size of 50 ms was then applied to further smooth the signal and reduce short-term fluctuations. Finally, the data were standardized to ensure consistent scaling across channels and subjects. No additional feature extraction was performed. The standardized time-series samples from the selected SDD channels within the 7 s analysis window were directly used as inputs to the classifiers.

#### 2.6.2. Motion Classification

Machine learning methods such as Support Vector Machine (SVM), Artificial Neural Networks (ANNs), and Linear Discriminant Analysis (LDA) have been widely used for motion recognition based on biosignals [30,31,32]. In this study, four models (SVM, Random Forest, ANN, and LDA) were evaluated using the preprocessed signals. All models were implemented using the scikit-learn library with default hyperparameter settings. Specifically, SVM was implemented using the default radial basis function kernel, Random Forest used the default ensemble configuration, ANN was implemented as the default multilayer perceptron classifier, and LDA was implemented using the default solver without shrinkage. The classifiers were trained using the preprocessed training data in each fold and evaluated on the held-out test sets.

Among these models, LDA achieved the highest classification accuracy and was selected as the final model for analysis. The classifier input consisted of the preprocessed six-channel time-series signals described above.

To ensure robust performance evaluation and reduce data bias, a five-fold cross-validation scheme was employed. A complete motion set, consisting of all nine motions performed sequentially, was treated as an independent classification unit. The dataset comprised 15 motion sets per subject, which were randomly divided into five groups containing three sets each. In each fold, one group (3 sets) was used as the test dataset, while the remaining four groups (12 sets) were used for training. Consequently, all samples belonging to a given set were assigned exclusively to either the training or testing partition, preventing overlap between temporally related samples and avoiding data leakage. This process was repeated across all folds, and the average classification accuracy was reported.

#### 2.6.3. Ablation Study

To evaluate the contribution of individual SDD channels to motion classification performance, an ablation study was conducted. All possible channel combinations from the six SDD channels were evaluated using the same preprocessing pipeline, LDA classifier, and five-fold set-level cross-validation procedure described above.

A total of 63 channel combinations were tested, ranging from single-channel configurations to the full six-channel configuration. For each combination, classification accuracy was calculated for all subjects, and the average accuracy across subjects was used for comparison. In addition, motion-specific F1-scores were analyzed to investigate the influence of different SDD combinations on individual forearm movements.

This analysis was performed to assess whether combining channels with different depth sensitivities provides additional discriminative information compared with individual channels or subsets of channels.

## 3. Results

This section presents the results of the depth sensitivity evaluation, followed by the results of the forearm motion classification.

### 3.1. Evaluation of Depth Sensitivity

**Figure 6:**
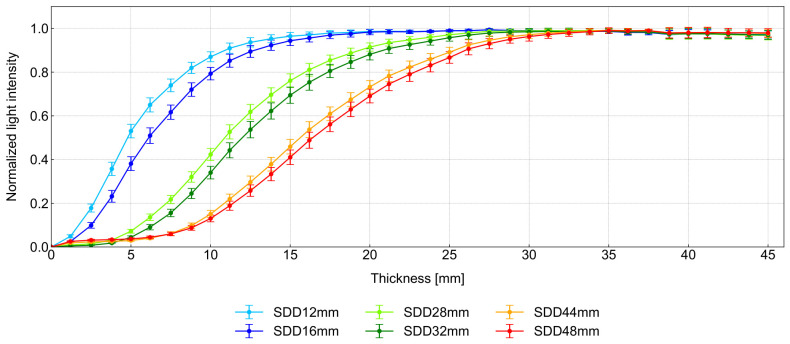
Result for the evaluation of the measurement depth.

The results of the depth sensitivity evaluation are shown in Figure 6, where the horizontal axis represents the thickness of the experimental medium and the vertical axis represents the normalized light intensity. Each data point corresponds to the measured light intensity at a given thickness for the six SDD channels, with error bars indicating the standard deviation across repeated trials.

Changes in light intensity with respect to thickness indicate that photons are reaching the absorbing layer (IR1500), whereas regions with minimal variation suggest that the medium thickness exceeds the effective penetration depth for the corresponding SDD. Based on these observations, the depth sensitivity for each SDD can be characterized as follows:

- SDD 12 mm and 16 mm: Significant changes in light intensity occur within approximately 2mm to 10 mm, with gradual variation up to around 20 mm. Beyond this range, the signal remains relatively constant.
- SDD 28 mm and 32 mm: Pronounced variation is observed within approximately 6 mm to 18 mm, with detectable sensitivity extending up to around 27 mm.
- SDD 44 mm and 48 mm: Light intensity varies over a broader range, approximately 10 mm to 25 mm, with sensitivity extending to around 32 mm.

These results indicate that increasing the SDD shifts the sensitivity of the measurement toward deeper regions. Additionally, shorter SDDs exhibit sharper changes over shallow depths, while longer SDDs show more gradual variations across a wider depth range, reflecting broader sensitivity profiles.

The estimated depth ranges are consistent with both the simulation results and approximate anatomical muscle depths, supporting the validity of the proposed approach. While the evaluation was conducted using a simplified phantom, the results demonstrate the underlying depth-dependent behavior of the sensor. In practical applications, additional physiological factors such as blood flow and tissue heterogeneity may influence the signal. Therefore, the reported depth ranges should be interpreted as approximate indicators of relative depth sensitivity rather than exact physiological depths in human tissue. Furthermore, signals measured at larger SDDs reflect contributions from multiple depth layers, providing depth-dependent information rather than isolating individual muscles.

### 3.2. Forearm Motion Classification

**Figure 7:**
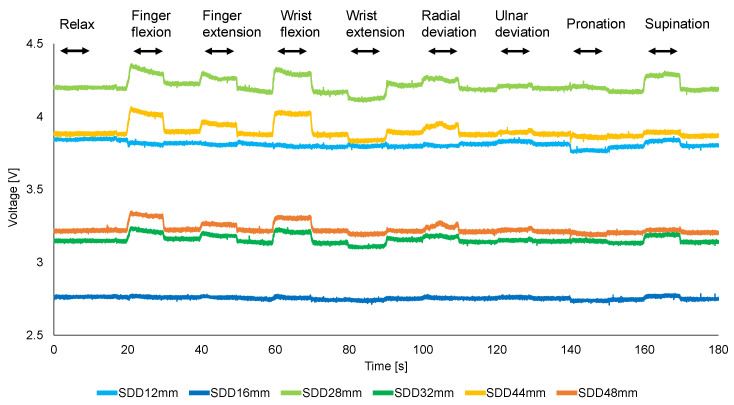
Raw signal of the sensor.

Figure 7 shows a representative example of raw signals from a single subject, illustrating light intensity variations measured by photo-detectors at different SDDs during nine forearm movements. The vertical axis represents voltage, which is proportional to the detected light intensity. Distinct signal patterns are observed across different movements.

Table 4 summarizes the F1-scores for each motion across all subjects. Wrist flexion and wrist extension achieved the highest classification performance, while the relax condition exhibited the lowest F1-score. Table 5 presents the overall classification accuracy for each subject. The highest accuracy was achieved by Subject 7 (96.3%), whereas Subject 4 showed the lowest performance (76.5%) among the participants.

Using all six SDD channels, the proposed sensor achieved an average classification accuracy of 87.5% across the nine forearm motions. These results demonstrate that the developed sensor can reliably distinguish multiple forearm movements using a single sensing module.

**Figure 8:**
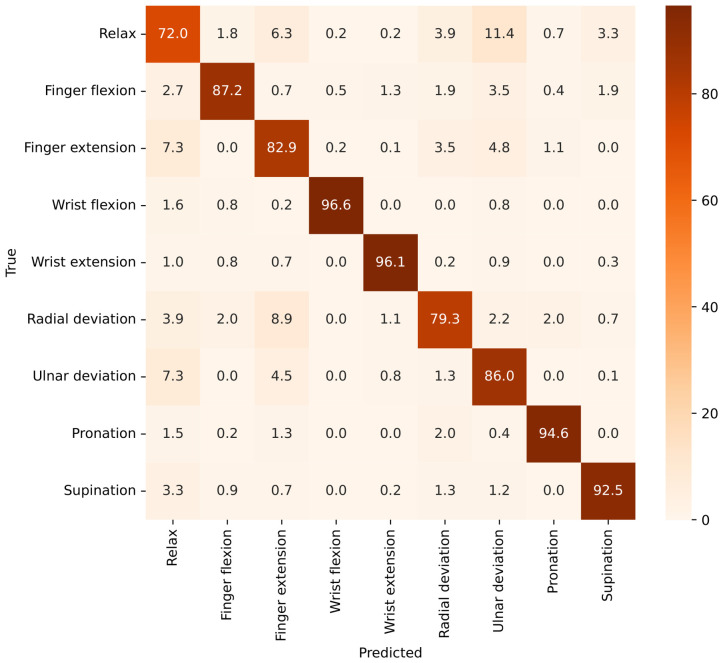
Normalized confusion matrix obtained using the six-channel configuration.

Figure 8 shows the normalized confusion matrix obtained using the six-channel configuration. Wrist flexion, wrist extension, pronation, and supination exhibited the highest classification rates, exceeding 92%. In contrast, the relax condition showed the lowest classification performance and was occasionally confused with finger extension, radial deviation, and ulnar deviation. Similar confusion was observed between finger extension and the relax condition. These results suggest that motions producing relatively small optical signal changes are more difficult to distinguish from resting states, whereas movements involving larger muscle activation patterns can be classified more reliably.

### 3.3. Ablation Study of SDD Channels

To investigate the contribution of individual SDD channels, an ablation study was performed using all 63 possible channel combinations. Table 6 summarizes the best-performing combination for each channel count.

Classification accuracy increased consistently as additional channels were incorporated, rising from 41.9% for the best single-channel configuration to 87.5% when all six channels were used. The optimal single-channel configuration was CH2, while the best two-channel configuration consisted of CH1 and CH4.

Notably, the optimal three-channel configuration consisted of CH1, CH5, and CH6 rather than adjacent channels with similar SDDs. Furthermore, CH5 and CH6 were retained in all optimal channel combinations containing three or more channels. If classification performance were determined solely by the number of sensing channels, combinations composed of neighboring SDDs would be expected to perform similarly. However, the observed preference for channels spanning different SDDs suggests that measurements acquired at different sensing depths provide complementary information for motion discrimination.

To quantitatively evaluate the effect of channel configuration, a Friedman test was performed using the best-performing combination for each channel count. The analysis revealed a significant effect of channel configuration on classification accuracy ($\chi^{2}\left(5\right)=48.4$, p<0.001). Post hoc Wilcoxon signed-rank tests further showed that the six-channel configuration significantly outperformed the best one-channel (p=0.00195), two-channel (p=0.00195), three-channel (p=0.00195), four-channel (p=0.00195), and five-channel (p=0.01953) configurations. These results provide statistical evidence that combining measurements from multiple SDDs significantly improves motion classification performance.

## 4. Discussion

Traditional non-invasive muscle activity measurement techniques capture cumulative signals from multiple muscle layers beneath the measurement site, limiting their ability to resolve depth-dependent information. While computational approaches can estimate depth-related features, they rely on indirect inference and are sensitive to modeling assumptions. In contrast, the proposed sensor introduces depth-dependent sensitivity at the hardware level by modulating the SDD. Although the complex anatomy of human muscles prevents direct mapping of signals to specific depths, the results demonstrate that the sensor captures depth-sensitive signal variations that can be exploited for motion discrimination.

The phantom experiment confirmed that increasing the SDD shifts the sensitivity of the measurement toward deeper regions. While the layered ham phantom does not fully replicate in vivo physiological conditions, it provides a controllable medium for evaluating depth-dependent optical behavior. The observed trends, where shorter SDDs are sensitive to shallow regions and longer SDDs capture signals influenced by deeper structures, are consistent with the Monte Carlo simulation results. Together, these findings support the validity of the proposed depth-sensitive sensing approach.

In the motion classification experiment, variations in performance across movements and subjects provide further insight into the sensing mechanism. Movements associated with larger or more distinct muscle activations generally achieved higher classification accuracy, likely due to more pronounced signal variations across multiple SDD channels. In contrast, the relax condition consistently exhibited lower performance, which can be attributed to minimal signal variation and overlap with low-amplitude signals from other movements. Confusion matrix analysis showed that the relax condition was most frequently confused with finger extension, radial deviation, and ulnar deviation, which also produced relatively small signal changes. Since reliable detection of the resting state is important for practical HMI and prosthetic-control applications, improving rest-state discrimination remains an important direction for future work.

For certain motions, such as supination and ulnar deviation, lower classification performance was observed in some subjects. This may be related to sensor placement, as the sensor was positioned on the flexor side of the forearm and therefore located farther from the primary muscles responsible for these actions. Despite this limitation, the sensor was still able to discriminate extension-related motions with relatively high accuracy. This suggests that the measured optical signals may be influenced not only by directly activated muscles but also by broader physiological effects, such as tissue deformation, co-contraction of antagonist muscles, and contributions from adjacent or deeper muscle groups. Therefore, the measured signals should not be interpreted as direct measurements of individual muscle activity. Rather, they reflect motion-related physiological changes within the forearm that are associated with the performed movement.

These results highlight a potential advantage of the proposed approach: the ability to extract informative motion-related patterns from a single sensing location. By capturing depth-dependent signal variations, the sensor may provide additional discriminative information without requiring multiple sensor placements, which is beneficial for wearable HMI applications where simplicity and compactness are essential.

The classification performance achieved in this study is comparable to existing non-invasive approaches for forearm motion recognition. Previous studies using sEMG-based systems have reported classification accuracies typically ranging from 80% to 95%, depending on the number of sensors, feature extraction methods, and classification models [30,31,32]. Similarly, high-density EMG and multi-sensor approaches can achieve high accuracy but often require complex hardware configurations and increased computational cost. In contrast, the proposed method achieves an average accuracy of 87.5% using a single optical sensor module, while additionally providing depth-dependent information. Although direct comparison is limited due to differences in experimental protocols and datasets, these results demonstrate that the proposed approach offers competitive performance while maintaining a compact and non-invasive design.

The ablation study further supports the proposed depth-sensitive sensing concept. While classification accuracy generally improved with increasing channel number, the optimal intermediate configurations consistently included deeper-sensitive channels (CH5 and CH6). In particular, the best three-channel configuration consisted of CH1, CH5, and CH6, suggesting that measurements obtained at different sensing depths provide complementary information for motion discrimination. These findings support the use of multi-SDD optical sensing for forearm motion recognition.

Additionally, variability across subjects was observed, reflecting differences in musculoskeletal structure, such as muscle geometry, positioning, and individual movement patterns. Classification accuracy varied across subjects, ranging from 76.5% to 96.3%. Subject 4 exhibited notably lower performance for finger extension and radial deviation, suggesting greater overlap between these motion patterns in this participant. This observation highlights the influence of individual differences in forearm anatomy, muscle geometry, tissue composition, sensor placement, and movement execution on the measured optical signals.

However, several limitations should be noted. First, the measured signals reflect a combination of multiple physiological factors, including tissue deformation, density changes, and hemodynamic effects, which are not individually separated in this study. Second, the phantom experiment provides an approximate evaluation of depth sensitivity and does not fully represent in vivo conditions. In addition, the Monte Carlo simulation was used to evaluate relative depth-dependent photon distributions rather than normalized sensitivity profiles. Consequently, quantitative metrics such as peak sensitivity depth, full width at half maximum (FWHM), and layer-specific sensitivity contributions were not extracted in the present study. Third, signals measured at larger SDDs include contributions from multiple tissue layers, limiting precise isolation of individual muscles.

Furthermore, no direct physiological ground-truth measurements, such as intramuscular EMG, ultrasound imaging, force/torque sensing, or NIRS, were acquired simultaneously. Therefore, the interpretation of depth-dependent muscle activity is supported indirectly through the combination of Monte Carlo simulations, phantom experiments, anatomical considerations, and motion-classification performance. Finally, inter-subject variability indicates that anatomical differences can influence signal characteristics, which may affect generalization performance.

Future work will focus on improving depth selectivity through optimized sensor configurations and advanced signal processing techniques. Validation using more physiologically accurate models and multimodal sensing approaches, such as combining optical sensing with ultrasound or EMG, could further enhance interpretability. Additionally, extending the system to multiple sensor placements may improve classification performance for a wider range of movements.

Overall, the results demonstrate that the proposed sensor can capture depth-dependent signal variations that are useful for motion classification, while maintaining a compact, non-invasive design suitable for wearable human–machine interface applications.

## 5. Conclusions

This study presented a novel optical sensing approach for the non-invasive measurement of muscle activity using multiple SDDs. By modulating measurement sensitivity through SDD, the proposed method introduces a simple yet effective means of incorporating depth-dependent information at the hardware level, addressing a key limitation of conventional non-invasive techniques that primarily capture cumulative surface signals.

Simulation and experimental evaluations demonstrated that signals obtained at different SDDs exhibit distinct depth sensitivities, consistent with both modeled light propagation and anatomical muscle structure. Leveraging these depth-dependent signal variations, the proposed sensor enabled accurate classification of multiple forearm motions using a single sensing module, achieving an average accuracy of 87.5% across ten subjects.

While the measured signals reflect a combination of physiological factors and precise mapping to specific muscle depths remains challenging, the results confirm that depth-sensitive information can be effectively captured and utilized for motion recognition. This highlights the potential of the proposed approach to enhance signal discrimination without increasing system complexity.

Future work will focus on improving depth selectivity, refining signal interpretation, and validating the approach using more physiologically representative models and multimodal sensing. Extending the system to multiple sensor placements, including both flexor and extensor regions, may further improve performance and robustness.

Overall, the proposed method provides a compact, non-invasive solution for integrating depth-sensitive sensing into wearable human–machine interface systems, with promising applications in prosthetics, rehabilitation robotics, and intuitive human–machine interaction.

## Figures and Tables

**Table 1:** Comparison of representative muscle sensing approaches for motion recognition.

Method	Wearable	Motion Classification	Depth-Dependent Information	Multi-SDD
sEMG [11,12]	√	√	×	×
MMG [5]	√	√	×	×
FMG [6]	√	√	×	×
HD-EMG [15,16]	Δ	√	×	×
Optical sensing [24,25]	√	√	Limited	×
Proposed method	√	√	√	√

**Table 2:** Forearm muscle depth.

	FCR	FDS	FDP
Subject 1	3–10 mm	10–18 mm	18–25 mm
Subject 2	4–11 mm	11–20 mm	20–27 mm
Subject 3	5–12 mm	12–20 mm	20–28 mm

**Table 3:** Primary muscle involvement during the nine forearm movements. ∘ indicates the muscle is expected to act as a primary agonist during the movement, whereas • indicates the muscle primarily acts as an antagonist or exhibits opposing activity.

	Relax	FF	FE	WF	WE	RD	UD	PRO	SUP
FCR				∘	•	∘	•		
FDS		∘	•	∘	•				
FDP		∘	•	∘	•				
PT								∘	•

**Table 4:** F1-scores for the nine forearm motions [%].

	Relax	FF	FE	WF	WE	RD	UD	PRO	SUP
Sub. 1	57.9	94.5	98.8	100.0	98.8	99.8	87.4	100.0	63.2
Sub. 2	85.4	91.5	95.8	94.6	98.2	88.8	88.8	99.3	99.9
Sub. 3	76.6	96.5	69.3	99.2	93.5	63.3	69.4	99.8	100.0
Sub. 4	59.8	71.7	55.4	91.4	95.5	61.8	63.3	100.0	82.6
Sub. 5	58.1	89.8	72.6	96.1	93.8	69.5	76.5	63.4	91.7
Sub. 6	71.3	96.1	79.6	100.0	98.1	88.4	72.3	100.0	96.3
Sub. 7	89.1	95.1	94.8	100.0	96.5	95.6	96.9	98.1	100.0
Sub. 8	72.1	96.4	81.1	100.0	100.0	69.8	81.4	99.5	95.3
Sub. 9	78.1	65.6	90.6	98.9	87.2	75.5	90.4	90.3	100.0
Sub. 10	68.3	97.0	64.7	95.8	99.3	97.9	90.8	100.0	99.7
Ave.	71.7 ± 11.0	89.4 ± 11.3	80.3 ± 14.7	97.6 ± 3.0	96.1 ± 3.8	81.0 ± 14.7	81.7 ± 11.0	95.0 ± 11.5	92.9 ± 11.8

**Table 5:** Average classification accuracy of each subject [%].

	Sub. 1	Sub. 2	Sub. 3	Sub. 4	Sub. 5	Sub. 6	Sub. 7	Sub. 8	Sub. 9	Sub. 10	Average
Accuracy	89.1	93.7	85.7	76.5	78.6	89.0	96.3	88.4	86.9	90.5	87.5 ± 6.1

**Table 6:** Best-performing channel combinations obtained from the ablation study.

Number of Channels	Best Channel Combination	Accuracy [%]
1	CH2	41.9±9.8
2	CH1 + CH4	65.5±8.6
3	CH1 + CH5 + CH6	77.3±9.9
4	CH1 + CH4 + CH5 + CH6	83.3±8.4
5	CH1 + CH2 + CH3 + CH5 + CH6	86.2±6.4
6	CH1 + CH2 + CH3 + CH4 + CH5 + CH6	87.5±6.1

## Data Availability

The data and analysis code are available from the corresponding author upon reasonable request.
